# Influence of Injection Molding Parameters on the Peel Strength between Plasma-Treated Fluoropolymer Films and Polycarbonate

**DOI:** 10.3390/polym15061568

**Published:** 2023-03-21

**Authors:** Martin Hubmann, Jonas Groten, Martin Pletz, Thomas Grießer, Kateřina Plevová, Wolfgang Nemitz, Barbara Stadlober

**Affiliations:** 1 Polymer Processing, Department of Polymer Engineering and Science, Montanuniversitaet Leoben, 8700 Leoben, Austria; 2 Joanneum Research Forschungsgesellschaft mbH, Franz-Pichler Str. 30, 8160 Weiz, Austria; 3 Designing Plastics and Composite Materials, Department of Polymer Engineering and Science, Montanuniversitaet Leoben, 8700 Leoben, Austria; 4 Chemistry of Polymeric Materials, Department of Polymer Engineering and Science, Montanuniversitaet Leoben, 8700 Leoben, Austria; 5 Materials Science and Testing of Polymers, Department of Polymer Engineering and Science, Montanuniversitaet Leoben, 8700 Leoben, Austria

**Keywords:** overmolding, simulation, cladding layer, THV, PC, peel test, XPS, AFM, light guiding

## Abstract

Light guiding is used to direct light from an emitting source to a different location. It is frequently realized through a clad–core structure with a difference in the refractive index of the materials. This paper explores the possibility of combining a fluoropolymer (THV) film of low refractive index, serving as a cladding layer, with a polycarbonate (PC) core, via injection molding. Pristine THV lacks adherence to the PC. However, when treated with $O_{2}$ plasma prior to overmolding, bonding can be established that was quantified in peel tests. The effect of this surface treatment was further investigated by adjusting the plasma treatment duration and time to overmolding. Furthermore, parameter studies comprising the four molding parameters, namely packing pressure, injection speed, melt temperature, and mold temperature, were performed. Numerical injection molding simulations assessed the prevailing temperatures at the PC–THV boundary. Consequently, the temperature–time integral could be calculated and linked with the measured peel strengths by fitting a proportionality constant. While the plasma treatment duration showed minor influence, the activation diminished with time, halving the measured peel loads within 24 h. The adhesion was experimentally found to increase with a lower packing pressure, faster injection speed, and higher melt and mold temperature. Those same molding relations influencing the peel loads were also found with the temperature–time integral when scaled by the proportionality constant in the simulations ($R^{2}=85\%$). Apparently, adhesion is added by molding settings which promote higher interface temperatures that prevail for longer. Hereby, the faster injection speed increases the melt temperature through shear heating. A higher packing pressure, in contrast, presumably increases the heat transfer at the PC–THV interface, accelerating the cooling. The measured peel loads were 0.3–1.6 N/mm for plasma-treated samples and nearly zero for pristine THV.

## 1. Introduction

In the in-mold decoration (IMD) process, a sheet or film is inserted into an injection mold and overmolded by rapidly injecting a thermoplastic melt. As a result, a plastic part with printed graphics and/or high gloss surfaces is manufactured [1]. This process can be adapted to the injection molded structural electronics (IMSE) process, where electronic components are integrated onto the films [2].

Today, IMSE is increasingly applied in human–machine interfaces (HMIs) where sensors and electronic connections are integrated into the injection molded parts. Those HMIs are fabricated as single plastic elements, reducing the weight and manufacturing time while increasing durability. Additionally, the design freedom is strongly increased, and backlighting touch keys and sliders became essential elements [2,3,4,5]. Usually, this is achieved by placing LEDs next to the areas to be illuminated. This can lead to the problem of substantial heat production close to the operation of the buttons. An alternative approach is to use light-guiding elements in such products, giving designers more freedom and solving the problem of local heat input. Here, the light could be directed from an emitting source, such as an LED, to an outcoupling area to be illuminated within a substantial distance from the LED, with the injection molded material serving as the light guiding plane—also called a waveguide. Two major elements facilitate this: the cladding layer must have a lower refractive index than the core (<). Then, the total reflection condition is fulfilled, and the light is “trapped” inside the waveguide [6,7]. A cladding layer can also act as a mechanical protection layer for the optical interface of the core.

This paper investigates the possibility of combining a fluorothermoplastic film containing tetrafluoroethylene, hexafluoropropylene, and vinylidene fluoride (THV) with a polycarbonate (PC) substrate using the IMD process. THV has a low refractive index ($n_{\mathrm{THV}}=1.36$ [8]) and could hence serve as a cladding layer for the PC core ($n_{\mathrm{PC}}=1.586$ [9]).

Proper bonding between the film and the overmolding material is crucial in the IMD process. The functionality and durability of the produced parts might not be guaranteed otherwise. Hence, research has been conducted to improve the adhesion between (thermo-plastic) films and molded parts by adjusting the molding settings:

Leong et al. [10] laminated semicrystalline, oriented polypropylene (PP), and cast PP films using polyurethane and subsequently overmolded them with PP. Above 180°, peel loads were obtained for the cast–PP–PP interface when the barrel temperature, injection speed, and packing pressure were increased. The fracture mode changed from peeling to film breakage depending on the used molding settings. Similar experiments were made by Hu et al. [11] with PP films overmolded with PP. Above 90°, peel loads were observed when the melt and mold temperature and the injection speed were increased. Likewise, thicker films yielded higher peel loads. Numerical simulations showed that higher interface temperatures and lower cooling rates prevailed. Again, different types of peel curves were obtained depending on the molding conditions. Based on differential scanning calorimeter (DSC) measurements, they concluded that the interface strength benefits from higher degrees of crystallinity.

In another report, Leong et al. [12] tested the bonding of semicrystalline polyethylene terephthalate (PET) films overmolded with PET. Above 180°, peel loads were recorded for film overmolding at higher melt temperatures and lower injection speeds. Different formations of crystallites were induced depending on the set injection speed, as shown by the DSC measurements of differently annealed samples. The authors concluded that highly oriented molecules—formed at higher injection speeds—exhibited fewer free molecules that could interact and form entanglements with the film interface. 

Finally, Leong et al. [13] examined amorphous PC films overmolded with amorphous PC acrylonitrile-butadiene-styrene (PC + ABS). Additionally, here thicker films showed peel loads above 90°. While a higher molecular weight of PC in the PC + ABS blend significantly enlarged the measured peel loads, the incorporation of PC-oligomers had the opposite effect.

Adding adhesive interlayers can facilitate the bonding of otherwise non-adhering film and overmolding material combinations [14,15]. Alternatively, adhesion promotor applications such as plasma or corona treatments might be used. Here, a material’s surface—but not the bulk—is modified. Frequently, polar functional groups are added to nonpolar substrates. In many cases, the hence “activated” surfaces show an “aging” effect—especially when stored in high-humidity environments—which means the treatment vanishes with time. The aging rate depends on the type of polymer and the storage conditions (humidity and temperature) [16]. Adjusting parameters such as the type of gas, gas flow, power, pressure, and treatment time makes it possible to tailor the treatment process to the used specimen. [16,17].

Ladner et al. [18] modified the surface of the nonpolar THV to incorporate different functional groups using plasma treatments and “click” chemistry processes. X-ray photoelectron spectroscopy (XPS) measurements showed the decrease in fluorine in their argon plasma pre-treated substrate. Furthermore, polar –OH and –COOH, and –COOR groups were formed.

Vasilets et al. [19] surface-functionalized polytetrafluorethylene (PTFE) using ${\mathrm{CO}}_{2}$ plasma activation and the vapor phase graft polymerization of acrylic acid (PAA). XPS measurements showed the presence of –COOR, –COH, and –C=O groups on the activated surface.

Barshilia et al. [20] surface-treated PTFFE with Ar + $O_{2}$ plasma. While there was no variation in the chemical structures observed, atomic force microscopy (AFM) images showed “leaf-like” micro-protrusions. This created a superhydrophobic surface with increased average surface roughness and a water contact angle (WCA) > 150°. Increases in the surface roughness and WCA of plasma-treated PTFE were also reported in [21,22].

**Figure 1:**
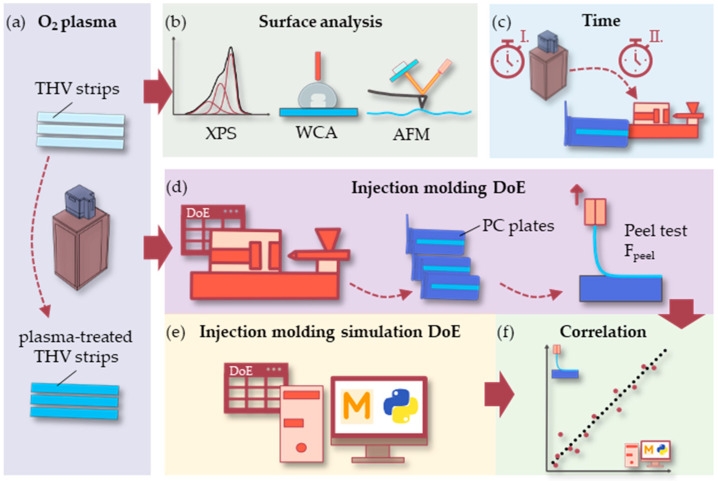
Schematic of the presented approach: THV strips were cut and plasma-treated (**a**). The as-modified surface was investigated by XPS, WCA, and AFM (**b**). Next, the influence of the plasma treatment time and the time between treatment and overmolding was investigated (**c**). Then, THV strips were overmolded with PC in a plate mold for subsequent peel testing. Furthermore, the effects of the molding parameters packed pressure, injection speed, melt, and mold temperature, which were investigated in a DoE (**d**). Finally, the DoE was numerically simulated using injection molding simulation (**e**). The aim was to find correlating quantities between the simulation and the peel test results (**f**).

To our knowledge, no studies in the literature are related to optimizing the injection overmolding of fluoropolymer films. Good adhesion between fluorinated and non-fluorinated materials is challenging but decisive in generating optical interfaces with high contrast in refractive index. In this work, THV strips were cut and surface-treated using $O_{2}$ plasma (Figure 1a). The modified surface was investigated using XPS, WCA measurements, and AFM (Figure 1b). The impact of the exposure time to the plasma and the time between treating the THV films and overmolding with PC was examined (Figure 1c). To that, the plasma-treated THV strips were overmolded in a plate mold. The PC–THV interface strength of the produced parts was assessed in peel tests. Similarly, a design of experiments (DoE) was performed to study the effects of the four molding parameters, namely the packing pressure, injection speed, melt temperature, and mold temperature (Figure 1d). The DoE was further numerically simulated using an injection molding simulation (Figure 1e) to find correlating quantities between the simulation and the peel test results (Figure 1f).

Finally, we demonstrated the benefit of adding a THV film as a scratch-protective cladding layer to a PC core in an overmolded waveguide structure.

## 2. Materials and Methods

### 2.1. THV Film Preparation and O_2_ Plasma Treatment

Films with a thickness of 0.2 mm out of THV GZ500 (3M Company, Saint Paul, MN, USA) were extruded by Maceplast GmbH (Jüchen, Germany) and were cut into 115 mm × 15 mm film strips and exposed to an $O_{2}$ plasma using an Oxford plasmalab system 80 plus (Oxford Instruments plc, Abingdon-on-Thames, UK). The treatment parameters are given in Table 1, and the exposure time was adapted as described in Section 2.6.

### 2.2. X-ray Photoelectron Spectroscopy (XPS) and Water Contact Angle (WCA)

The chemical surface compositions of the pristine (non-plasma-treated) and the $O_{2}$ plasma-treated (setting given in Table 1 with 2 min exposure time) THV films were analyzed by a thermoscientific Nexsa G2 (Thermo Fisher Scientific Inc., Waltham, MA, USA) XPS system. The instrument used a monochromatized Al Kα X-ray source. The analyzer operated with a pass energy of 20 eV and a step size of 0.100 eV. The ${–\mathrm{CF}}_{2}$ peak (292 eV [21]) was used for the calibration of the binding energy (BE) scale.

Likewise, the contact angles with distilled water (WCA) of the THV films were measured using a KRÜSS DSA100 (KRÜSS GmbH, Hamburg, Germany) drop shape analyzer (liq. vol. 2 µL, drops per setting *n* = 7).

### 2.3. Atomic Force Microscopy (AFM)

The topography of the pristine and $O_{2}$ plasmas-treated (setting given in Table 1 with 2 min exposure time) THV films were investigated on an AFM-IR VistaScope 75 (Anfatec Instruments AG, Oelsnitz/Vogtl, Germany). The probes were highly doped silicon tips with gold coating and force constants of 10–130 N/m, resonance frequencies lying in the 204–497 kHz range, and a typical radius of curvature <10 nm. The images were recorded with a resolution of 512 × 512 pixels. The AFM microscope was operated in photo-induced force mode.

### 2.4. Injection Molding 

**Figure 2:**
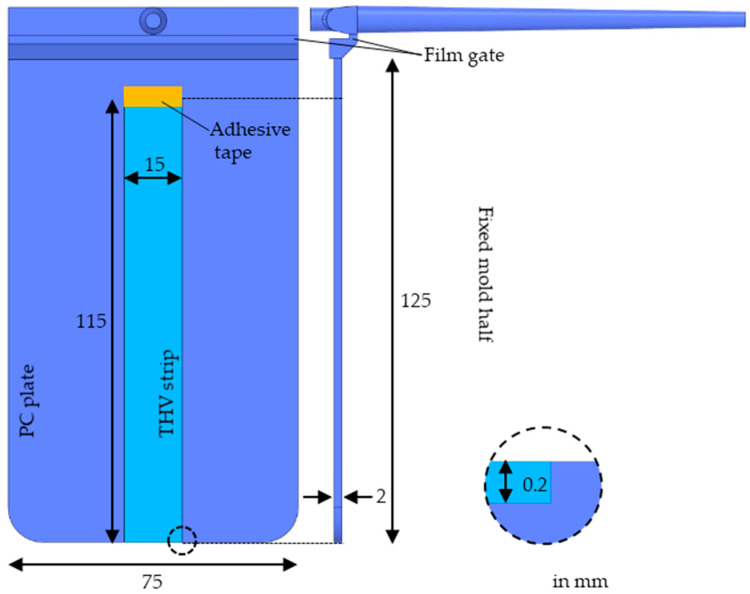
Dimension of the cut THV strips and injection molded PC plates.

The plasma-treated THV strips were then overmolded with the polycarbonate PC Lexan OQ1028 (Sabic, Riyadh, Saudi Arabia) of high-optical quality. To that end, the films were inserted into a rectangular 2 mm thick plate mold, as shown in Figure 2, and fixed by applying a temperature-resistant adhesive tape on the flow front-facing film side.

The parts were produced on a fully electric Arburg Allrounder 470 A Alldrive (Arburg GmbH + Co KG, Loßburg, Germany) injection molding machine with a 25 mm screw. A Wittmann Tempro plus D 160 (WITTMANN Technology GmbH, Wien, Austria) temperature control unit was utilized to heat the mold. The dosing volume was set to 40 cm^3^, and the switch-over point (velocity- to pressure-controlled filling) was adapted for each setting. The packing pressure was applied for 15 s, and the residual cooling time was set to 50 s. Process parameters that were adapted for the individual tests are given in Section 2.6.

### 2.5. Peel Test Analysis Procedure

**Figure 3:**
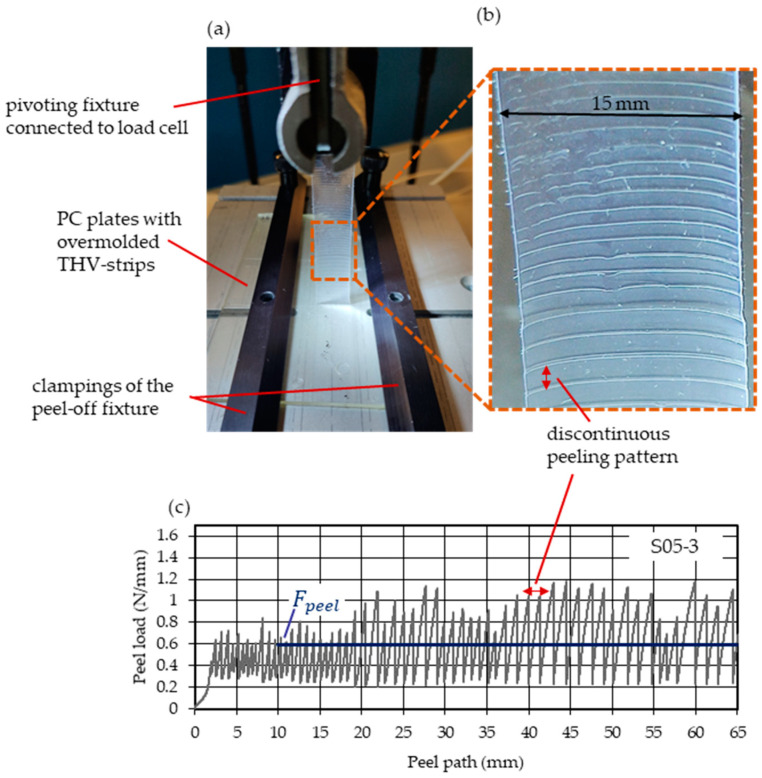
Peel test setup (**a**) and discontinuous peeling of THV strip (**b**) that resulted in an oscillating peel load curve (**c**) of which the mean peel load ($F_{\mathrm{peel}}$) was calculated (blue line).

The bonding strength between the THV strips and PC plates (Figure 2) was assessed via peel tests. To that end, an Instron 5500R (Illinois Tool Works Inc., Glenview, IL, USA) tensile test machine equipped with a peel-off fixture was used. First, the molded PC plates were positioned with the THV films along the centerline of the peel-off fixture. Next, two clampings were applied on both sides with a gap of ~10 mm to the THV films. The pivoting fixture then guaranteed the vertical alignment between the specimen and the fixture. An image of the test setup is given in Figure 3a.

The THV film strips were peeled at 90° with a 10 mm/min peel speed for a distance of 55 mm. 

The force—recorded using a 100 N static load cell at a frequency of 10 $s^{-1}$—was divided by the film’s width (15 mm) to yield the peel loads in N/mm. 

A pronounced discontinuous peeling was observed (“stick–slip” effect) as displayed for a peeled THV strip in Figure 3b. In addition, it resulted in oscillating peel loads increasing in amplitude with progressing peel length. For further comparisons, the mean peel load ($F_{\mathrm{peel}}$) was calculated as shown in Figure 3c.

### 2.6. Experimental Design

This section describes the parameters used for manufacturing the overmolded THV strips that were subsequently subjected to peel tests, as described in Section 2.5.

#### 2.6.1. Variation of Time of $O_{2}$ Plasma Exposure

The exposure time of the THV strips to the plasma was investigated at four intervals of 0.5 min, 1 min, 2 min, and 3 min. Four THV strips per investigated duration were overmolded 1 h after plasma treatment using the molding parameter S05.

#### 2.6.2. Variation of Time from $O_{2}$ Plasma Treatment to Overmolding

The influence of the period between plasma treatment and overmolding of the THV strips was investigated at five intervals of 1 h, 2 h, 3 h, 4 h, and 24 h. Four THV strips per investigated period were etched for 2 min and overmolded using the molding parameters S05. In addition, the WCA was measured at those intervals.

#### 2.6.3. Variation of Molding Parameters (Design of Experiment, DoE)

The influence of the molding parameters packing pressure (A), injection speed (B), melt temperature (C), and mold temperature (D) on the peel loads ($F_{\mathrm{peel}}$) was investigated using a 2-level full factorial design of experiments (DoE).

In such parameter studies, all possible factor (*k*) combinations at two set levels (low and high) are realized, resulting in a total of $2^{k}$ different settings. Additionally, a center point (CtPt) was added in which all the factors are set to the intermediate level (S05). The used DoE is given in Table 2.

The THV strips were plasma treated under a $O_{2}$ environment using the setting described in Table 1 with an exposure time of 2 min. Three parts per setting were produced, which resulted in a total of 3 × (16 + 1) = 51 runs.

The DoE was analyzed using the statistic software Minitab (Minitab Inc., State College, PA, USA). A linear regressions model linking the investigated input variables (factors A–D) via the associated coefficients with the peel load ($F_{\mathrm{peel}}$) was set up.

The model’s input variables’ statistical significances were assessed through the analysis of variance (ANOVA). As part of such an analysis, individual *p*-values are calculated for each model’s coefficients. The *p*-value indicates the risk of rejecting the null hypothesis (no relationship between factor and response) when the null hypothesis is true. Frequently a factor is considered significant (and not of random origin) for *p* ≤ *α* = 0.05, with *α* designating the significance level [23].

Models based on 2-level designs inevitably assume a linear relationship between the input variables and the response. However, possible nonlinearity (within the design space) can be detected by including a designated center point term (CtPt) in the model and evaluating its *p*-value.

### 2.7. Injection Molding Simulation of DoE

The commercial injection molding simulation software Autodesk Moldflow Insight 2021 (AMI, Autodesk Inc., San Rafael, CA, USA) was used to numerically study the 17 settings of the DoE (Table 2). It provides insight into the temperature evaluation of the PC–THV boundary. Consequently, simulation can help in developing a more profound process understanding. This section describes the created AMI model (Section 2.7.1), followed by a description of the analysis procedure performed (Section 2.7.2). Here, attempts were made to correlate the simulation results with the measured peel loads ($F_{\mathrm{peel}}$) of the DoE described in Section 2.5.

#### 2.7.1. Simulation Model Preparation

AMI numerically solves the conservation equations of mass, momentum, and energy to model the filling and packing phase (which AMI extends by the selected cooling time) using the finite element method (FEM) [24].

A 3D FEM model was created featuring the THV strip (AMI property part insert) and the rectangular 2 mm thick plate (AMI property part), as shown in Figure 2. The global edge length was set to 1 mm with a minimum number of 12 elements through the thickness of the injection molded plate. The film gate and the region of the part in contact with the THV strip were modeled with a mesh size of 0.5 mm. A minimum number of 12 elements through the thickness and a mesh size of 0.5 mm were selected for the THV strip. The auto-sizing scale factor was set to 0.9, and the machine die was modeled as a beam hot runner.

The linear tetrahedral element count for the model was 2 259 289/910 426 (plate/THV strip, respectively).

The simulation material data (such as Cross-WLF viscosity coefficients and Tait pvT coefficients) for the PC Lexan OQ1028 overmolding material were provided by Sabic (Autodesk udb-file).

**Figure 4:**
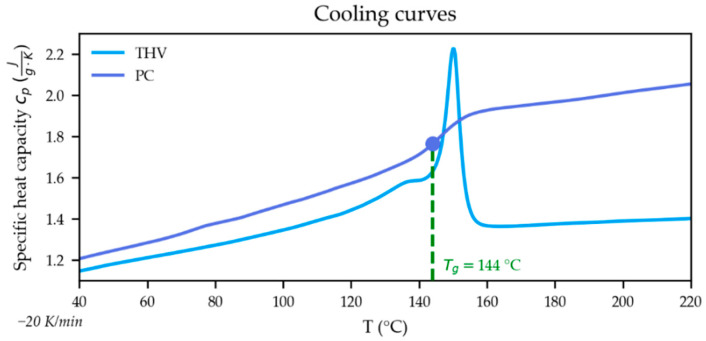
Specific heat capacity curves of the used THV and PC. The glass transition temperature (Tg) of the PC is additionally marked.

The specific heat capacity ($c_{p}$) of the THV and the PC were measured using a differential scanning calorimeter DSC1 (Mettler-Toledo International Inc., Columbus, OH, USA) at a cooling rate of −20 K/min and are plotted in Figure 4 together with the glass transition temperature Tg of the PC. 

Values from the literature were used for the thermal conductivity (λ=0.202 W/(m·K) [25]) and density ($\rho =2.01\;g/{\mathrm{cm}}^{3}$ [8]) of the THV, which were simplified to be constant.

In AMI, different heat transfer coefficients (HTCs) can be assigned to the molding cycle’s filling, packing, and detached (pressure is zero) phases. Table 3 states the AMI default HTC values used for the PC–THV interface.

A constant mold wall temperature was assumed as the surface boundary condition and set according to the experimental design (Table 2).

A starting temperature of 25 °C was assigned to the THV strip, and a 10 s contact time with the hotter mold before the start of injection was specified. This should be about the time it took the operator to start a new injection molding cycle after inserting the THV strip into the mold.

Fill + pack was selected as the analysis sequence, with the solver set at maximum %volume to fill per time step adapted to 2% and the maximum packing time step set to 0.5 s. In addition, to portray the process conditions at a higher time resolution, 10 intermediate results from the moment the PC melt contacts the THV strip until the end of the filling and 20 intermediate results for the first 12 s of the packing phase were selected.

#### 2.7.2. Simulation Analysis Procedure

The numerically simulated DoE was examined in view of the experimentally obtained peel loads ($F_{\mathrm{peel}}$) to find possible correlations. Therefore, a closer look was taken at the results of the part nodes (PC plate) in contact with the modeled THV strip. 

The following quantities at this PC–THV boundary were investigated:

1. $f_{T}$—temperature at flow front;
2. $f_{t}$—time the PC melt is above its Tg;
3. $f_{A}$—temperature–time integral while the PC melt is above its Tg, defined as
  (1) $$f_{A}=\int_{0}^{f_{t}}\left(T(t)-T_{g}\right)\mathrm{dt}$$

**Figure 5:**
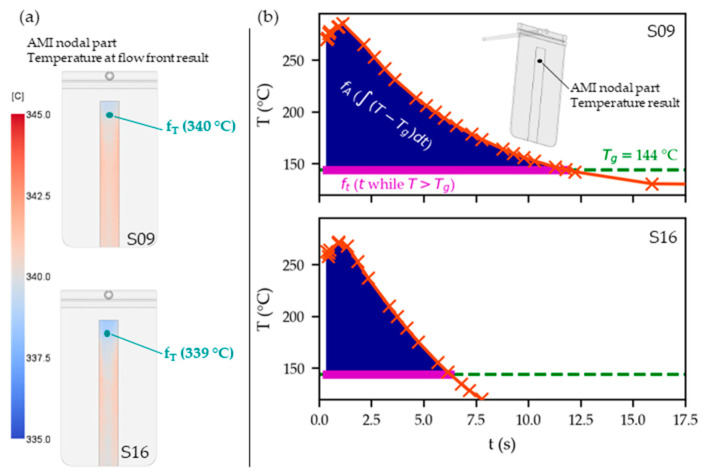
Contour plot of the “temperature at flow front result” ($f_{T}$) at the PC–THV boundary for settings S09 and S16 (**a**). A schematic representation of $f_{t}$ (time PC melt is above its Tg in magenta and $f_{A}$ (temperature–time integral as defined in Equation (1)) in blue is shown for one node for settings S09 and S16 (**b**).

The “temperature at flow front result” ($f_{T}$) is a default result in AMI that is hence easy to obtain and to evaluate immediately. It shows the temperature of the polymer when the flow front reaches a specified point in the center of the plastic cross-section [26]. Figure 5a shows shaded contour plots of this result for S09 and S16 at the examined PC–THV boundary.

In contrast, the AMI default result, “temperature result”, yields the temperatures at a specified time [27]. It is exemplarily displayed for one part node at the PC–THV boundary in Figure 5b for S09 and S16. Based on these results, the nodal $f_{t}$ and $f_{A}$ can be derived as shown in magenta and blue in Figure 5b, respectively.

A Python script was developed that made use of the synergy application programming interface (API) [28] to extract the nodal $f_{T}$ results at the PC–THV boundary. Likewise, individual nodal $f_{t}$ were calculated using the Python scipy.integrate.root_scalar routine [29] to numerically find the point in time when the PC melt cools below $T_{g}$. Based on these, individual nodal $f_{A}$ could be calculated by applying the Python scipy.integrate.quad numerical integration routine [30] on Equation (1).

The (nodal-)average values $\bar{f_{T}}$, $\bar{f_{t}},$ and $\bar{f_{A}}$ were then computed per setting (S⋮) of the DoE. Proportionality between those quantities and the measured peel strength (${F_{\mathrm{peel}}}_{S\vdots }$) of the corresponding setting was assumed with:

(2) $${F_{\mathrm{peel}}}_{S\vdots }=c_{\bar{f_{T}}}\cdot {\bar{f_{T}}}_{S\vdots }$$

(3) $${F_{\mathrm{peel}}}_{S\vdots }=c_{\bar{f_{t}}}\cdot {\bar{f_{t}}}_{S\vdots }$$

(4) $${F_{\mathrm{peel}}}_{S\vdots }=c_{\bar{f_{A}}}\cdot {\bar{f_{A}}}_{S\vdots }$$

respectively. The proportionality constants ($c_{\bar{f_{T}}}$, $c_{\bar{f_{t}}}$, and $c_{\bar{f_{A}}}$) were estimated using the Python scipy.integrate.curve fit routine [31].

## 3. Results and Discussion

### 3.1. X-ray Photoelectron Spectroscopy (XPS) Evaluation

**Figure 6:**
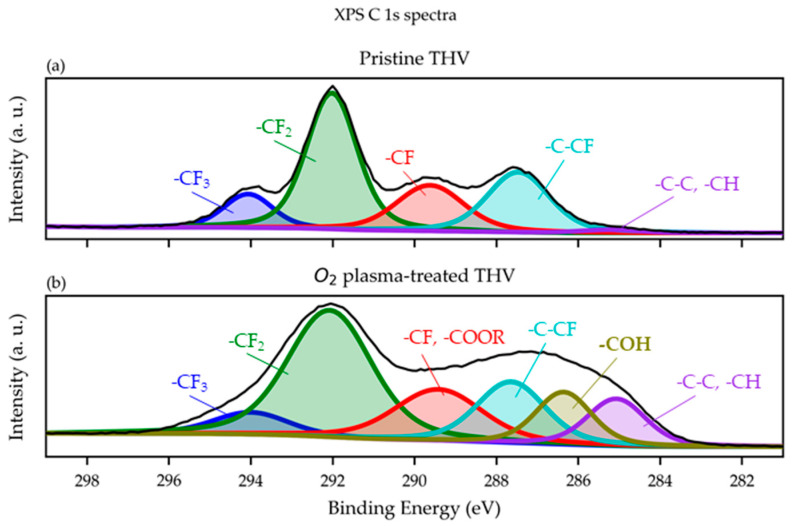
Comparison of the XPS C 1s spectra of pristine (**a**) and $O_{2}$ plasma-treated THV showing oxygen-containing –COH and –COOR surface groups (**b**).

The XPS C 1s spectra of pristine and $O_{2}$ plasma-treated (setting given in Table 1 with 2 min exposure time) THV are given in Figure 6a and Figure 6b, respectively.

The obtained XPS spectra show the presence of ${\text{–}\mathrm{CF}}_{3}$, ${\text{–}\mathrm{CF}}_{2}$, –CF, and –C–CF peaks, which are characteristic of the THV substrate [32,33,34]. The curve fitting for the high-resolution C(1s) peaks was determined using least-squares peak fitting. The C(1s) spectra of the non-modified THV surface (Figure 6a) were fitted with five spectral components assigned to –CH and –C–C (BE = 285.05 eV), –C–CF (BE = 287.50 eV), –CF (BE = 289.60 eV), –${\mathrm{CF}}_{2}$ (BE = 292.00 eV), and –CF_3_ (BE = 294.10 eV).

For the $O_{2}$ plasma-treated THV (Figure 6b), the C(1s) spectra were fitted with six spectral components assigned to –CH and –C–C (BE = 299.85 eV), –COH (BE = 286.35 eV); –C–CF (BE = 287.65 eV), –CF; –COOR (BE = 289.45 eV), ${–\mathrm{CF}}_{2}$ (BE = 292.05 eV), and ${–\mathrm{CF}}_{3}$ (BE = 294.05 eV). Interestingly, the $–{\mathrm{CF}}_{3}$ peak significantly decreases after plasma treatment, while an increase in the –CH peak is observed (Figure 6b). This can be explained by the defluorination of the THV surface [18], which is confirmed by a concomitant decrease (from 61.80% to 55.3%) in the overall fluorine content (see Table 4). In addition, the $O_{2}$ plasma treatment resulted in an increase in atomic oxygen concentration (from 0.0% to 2.5%, Table 4) due to the generation of oxygen-containing groups (–COH (BE = 286.35 eV) and –COOR (BE = 289.45 eV); Figure 6b).

The incorporation of oxygen groups by the $O_{2}$ plasma treatment is also apparent in Table 4, listing the surface element compositions.

### 3.2. Atomic Force Microscopy (AFM) Evaluation

**Figure 7:**
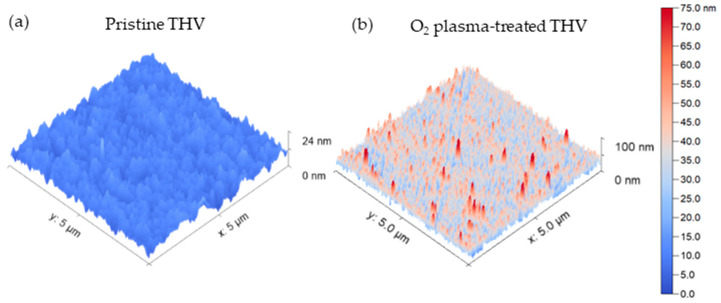
AFM images of the pristine (**a**) and $O_{2}$ plasma-treated THV films (**b**).

The AFM images for the pristine and $O_{2}$ plasma-treated (setting given in Table 1 with 2 min exposure time) THV films are given in Figure 7a and Figure 7b, respectively. They reveal a distinct increase in the nanoscale roughness due to the O2-plasma treatment, similarly to previous findings [20,21,22].

The root mean square roughness ($S_{q}$) for the samples shown in Figure 7 and calculated using the visualization software Gwyddion (accessed on 19 January 2023) [35], increased from 2.2 nm (pristine) to 9.8 nm (plasma-treated THV).

### 3.3. Peel Test Evaluation

This section presents and discusses the peel test results of the THV strips overmolded with PC using the settings described in Section 2.6.

#### 3.3.1. Study of Time of $O_{2}$ Plasma Exposure

**Figure 8:**
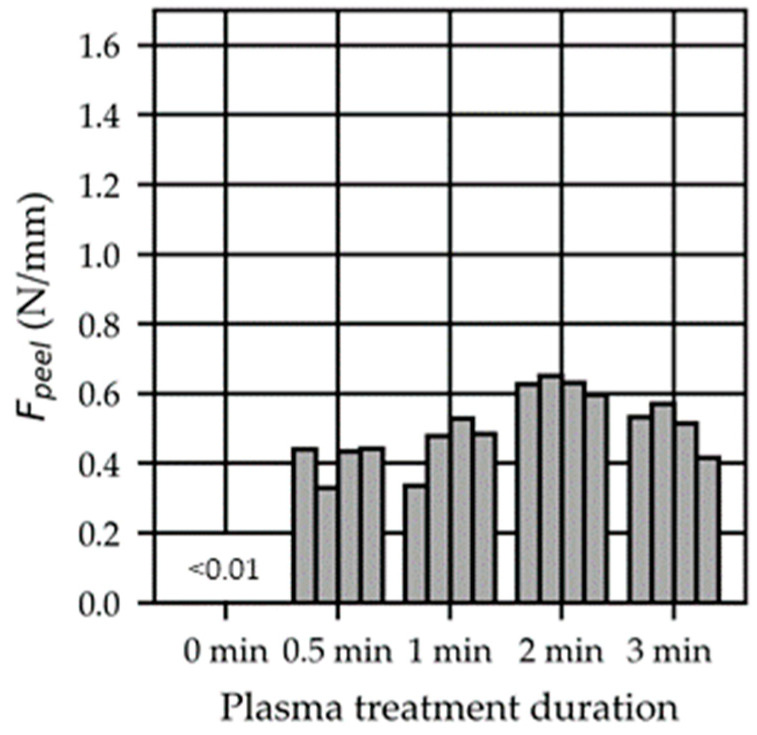
Influence of the peel load ($F_{\mathrm{peel}}$) on the plasma treatment duration. The THV strips were overmolded 1 h after plasma treatment using setting S05 (CtPt).

Pristine THV strips overmolded with PC were mostly separated upon demolding (“no adhesion”). Already a short 0.5 min $O_{2}$ plasma exposure of the THV facilitates bonding after overmolding, as shown in Figure 8. In our tests, a plasma treatment duration of 2 min yielded the highest peel loads ($F_{\mathrm{peel}}$).

#### 3.3.2. Study of Time from $O_{2}$ Plasma Treatment to Overmolding and Water Contact Angle (WCA)

**Figure 9:**
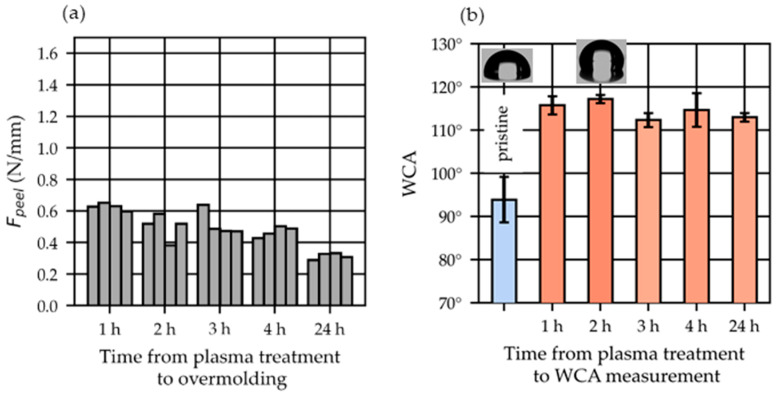
Influence of the time between plasma treatment and overmolding on the peel load ($F_{\mathrm{peel}}$, (**a**)) and on the water contact angle (WCA), shown as mean values ± standard deviation (*n* = 7, (**b**)). The THV strips were overmolded using setting S05 (CtPt) and were plasma-treated for 2 min.

The plasma surface activation of the THV strips diminished over time, as shown in Figure 9a. The THV strips investigated herein were plasma-treated under an $O_{2}$ environment using the setting described in Table 1 with an exposure time of 2 min before overmolding. The peel loads were about half as high for films overmolded 24 h after plasma treatment as they were for films that were overmolded 1 h after.

The water contact angle (WCA) increased considerably from ~94° for the pristine THV to ~115° right after $O_{2}$ plasma treatment is depicted in Figure 9b. The WCA remains constant during the investigated period (1–24) h after treatment.

The change in the wetting behavior was primarily attributed to the increase in the nanoscale roughness (compare Figure 7) [20,21,22].

#### 3.3.3. Study of Molding Parameters

**Figure 10:**
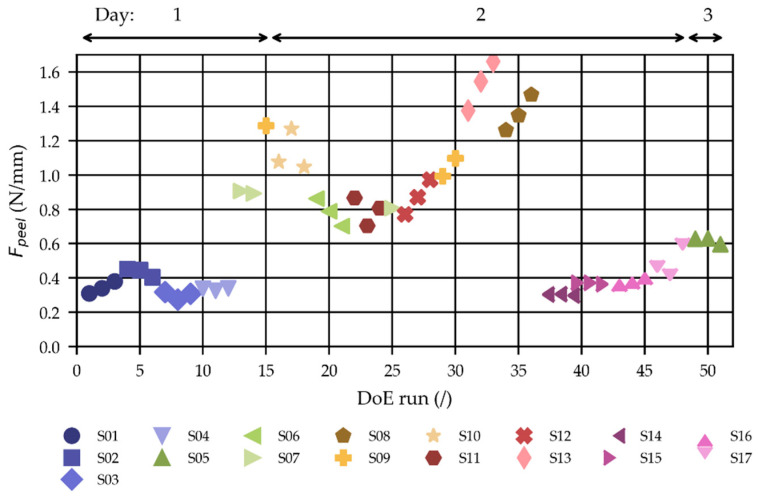
Obtained peel loads ($F_{\mathrm{peel}}$) of the DoE defined in Table 2 in molding order (3 parts/setting).

Figure 10 shows the obtained peel loads ($F_{\mathrm{peel}}$) of the DoE (Table 2) to investigate the impact of molding parameters. The THV strips investigated herein were plasma-treated under $O_{2}$ environment using the setting described in Table 1 with an exposure time of 2 min before overmolding.

The higher the peel loads, the stronger the scattering of the data. This is attributed to the more pronounced discontinuous peeling (more oscillating peel load curve) observed in those settings (see Figure 3).

The multiple comparisons method [36] was used to test whether the variances between the settings (with data displayed in Figure 10) are equal. At a *p*-value of p=0.002, below the frequently used significance level of α = 0.05, it cannot be concluded that all variances are equal. By performing a Box-Cox transformation [37] with

(5) $$Y=-\left({F_{\mathrm{Peel}}}^{-0.5}\right)$$

more uniform variances could be obtained. The multiple comparisons test for the transformed data (Y) was p=0.165. Hence, the ANOVA—in which variance homogeneity in the response is assumed [23]—was performed on the transformed data (Equation (5)) of the DoE (Table 2). Only the (stronger) main effects were considered, and Table 5 displays the *p*-values of the factors, which are all below α=0.05. This indicates their statistically significant influence. However, the *p*-value of the center points (CtPt) is large, meaning that it cannot be concluded that any of the factors have a curved relationship with the response.

The associated regression equation reads

(6) $$Y\;({\left(N/mm\right)}^{-0.5})=-4.1-4.8{\cdot 10}^{-4}\cdot A+2.5{\cdot 10}^{-3}\cdot B+5.5{\cdot 10}^{-3}\cdot C+1.1{\cdot 10}^{-2}\cdot D+5.8{\cdot 10}^{-2}\cdot \mathrm{CtPt}$$

where the units of A, B, C, D, and CtPt are the bar, ${\mathrm{cm}}^{3}/s$, °C, °C and 1, respectively. The CtPt variable is 1 for the center point setting (S05) and 0 for any other setting. Its coefficient of determination is $R^{2}=95.28\%$ (and adjusted coefficients of determination is ${R^{2}}_{\mathrm{adj}.}=94.76\%$).

**Figure 11:**
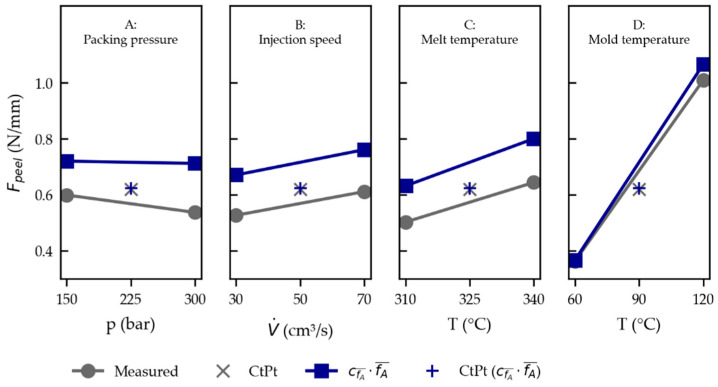
Effects plot based on the regression of the DoE for the measured peel loads given in Equation (6) in gray and for the simulated peel loads given in Equation (7) in blue, with CtPt designating the center point (S05).

The corresponding factorial plots that display the relationships between the response and the individual variables are plotted in Figure 11 in grey below. The calculated response is plotted in each plot when the investigated factor is set to its low and high level, while the other factors are set to the intermediate level. The diagrams visualize that a low packing pressure (A), high injection speed (B), high melt temperature (C), and—by far the most influential—high-mold temperature (D) result in a higher peel load ($F_{\mathrm{Peel}}$). 

The DoE was molded on three days, with the THV films plasma-treated at the start of the day. The time from surface activation to overmolding was between 1 and 3 h for the films due to the required waiting periods between temperature changes. However, as shown in Section 3.3.2, the plasma activation decreases during this time. Therefore, the results of the analysis are likely to be affected by this to some degree.

### 3.4. Analysis of Simulated DoE Concerning Peel Test Results

The values for the proportionality (“scaling”) constants defined in Equations (2)–(4) were estimated as:

1. $c_{\bar{f_{T}}}=2.15{\cdot 10}^{-3}\;N/(\mathrm{mm}\cdot K)$;
2. $c_{\bar{f_{t}}}=1.16{\cdot 10}^{-1}\;N/(\mathrm{mm}\cdot s)$;
3. $c_{\bar{f_{A}}}=3.38{\cdot 10}^{-3}\;N/(\mathrm{mm}\cdot K\cdot s)$.

**Figure 12:**
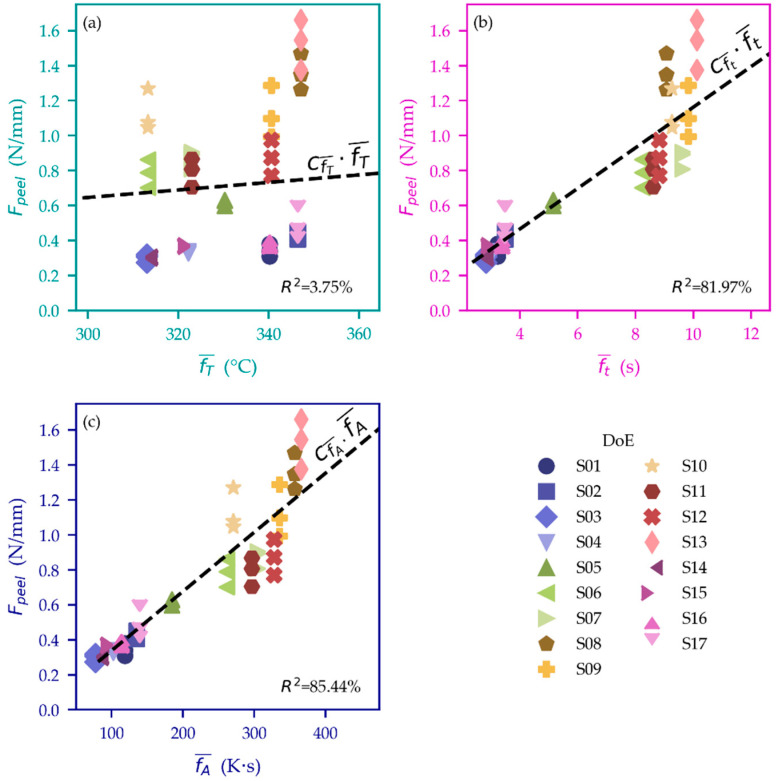
Comparison of the investigated simulation quantities (*x* axis) and measured peel loads according to Figure 10 (*y* axis): there is hardly any correlation between $\bar{f_{T}}$ (averaged temperature at the flow front) and the measured peel load, as indicated by the low $R^{2}$-value in (**a**). A better correlation can be seen for $\bar{f_{t}}$ (averaged time for which the PC–THV interface is hotter than $T_{g}$ ) in (**b**) and $\bar{f_{A}}$ as defined in Equation (1) in (**c**) (data points below and above the dashed line are over- and underestimated in the simulation, respectively).

Figure 12 shows comparisons of the investigated simulation quantities (x axis) with the measured peel loads ($F_{\mathrm{peel}}$, y axis) of the DoE (Table 2). There is no good correlation with the melt front temperature ($\bar{f_{T}}$, Figure 12a). A better correlation can be found when observing the averaged time where the PC–THV boundary is above $T_{g}$ ($\bar{f_{t}}$, Figure 12b). A further increase in the correlation (higher $R^{2}$-value) is observed when considering the temperature–time integral as defined in Equation (1) ($\bar{f_{A}}$, Figure 12c).

The regression using the temperature–time integral ($F_{\mathrm{Peel},\mathrm{pred}.}=c_{\bar{f_{A}}}\cdot \bar{f_{A}}$) fitted to the simulated DoE reads

(7) $$F_{\mathrm{peel}\;\mathrm{pred}.}\;(N/\mathrm{mm})=-2.3-5.5{\cdot 10}^{-5}\cdot A+2.3{\cdot 10}^{-3}\cdot B+5.6{\cdot 10}^{-3}\cdot C+1.2{\cdot 10}^{-2}\cdot D-9.2{\cdot 10}^{-2}\cdot \mathrm{CtPt}$$

where the units of A, B, C, D, and CtPt denote the bar, ${\mathrm{cm}}^{3}/s$, °C, °C, and 1, respectively ($R^{2}=99.48\%$). The CtPt variable is 1 for the center point setting (S05) and 0 for any other setting.

The corresponding factorial plots depicted in Figure 11 in blue show similar dependencies to those found in the experimental DoE in grey (compare Section 3.3.3).

A higher (set) melt temperature (C) and mold temperature (D) establishes a higher PC–THV interface temperature that cools down more slowly, apparently improving adhesion. Likewise, a faster injection speed (B) causes an increase in the melt temperature due to shear heating (viscous dissipation).

**Figure 13:**
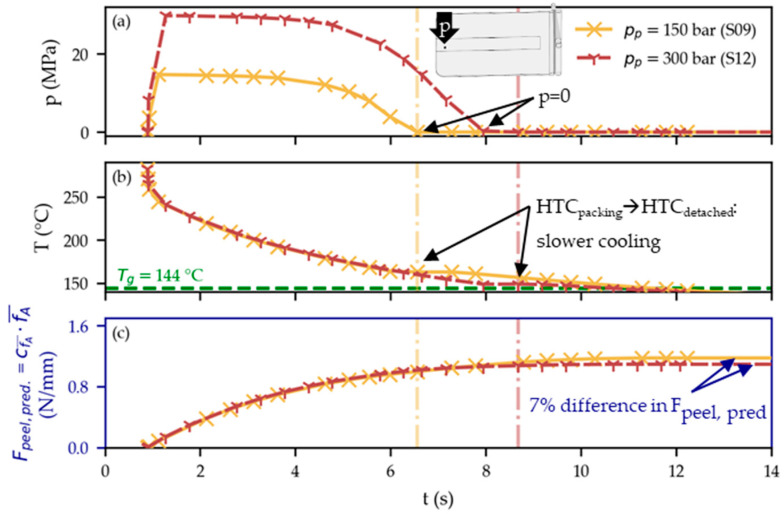
Comparison of S09 and S12 for a PC–THV boundary node. The settings only differ in the set packing pressure. The higher applied packing pressure of S12 results in longer cavity pressurization compared to S09 (**a**). Consequently, AMI uses the higher HTC_packing_ for longer before switching to the HTC_detached_ (values given in Table 3). Hence, the interface cools down quicker when a (packing) pressure is still prevailing in the mold (**b**) and the predicted peel load ($F_{\mathrm{peel}\;\mathrm{pred}.}$) is subsequently lower (**c**).

A lower packing pressure (A) was found to aid adhesion in the simulations, however, on a smaller scale. Presumably, a higher packing pressure evokes a larger heat transfer coefficient (HTC). The PC–THV interface hence cools quicker when pressure is prevailing. This can be seen in the simulation result—especially in areas farther from the gate where the difference in pressurization time between a set high and low packing pressure is more pronounced. Figure 13 contrasts S09 and S12 of the DoE, which only differ in terms of applied packing pressure (150 and 300 bar, respectively). The higher applied packing pressure of S12 results in a longer period of pressurization within the cavity (Figure 13a). AMI changes the HTC according to Table 3 when the pressure reaches zero, which has an impact on the calculated temperatures (Figure 13b) and, consequently, on the predicted peel loads $F_{\mathrm{peel}\;\mathrm{pred}.}$ (Figure 13c). In reality, there is no abrupt switch of the HTC as modeled in AMI. However, the pressure dependency of the HTC is generally known from the literature [38]. This would explain the experimentally found relation of a lower packing pressure (A) increasing the adhesion between the THV film and the PC substrate.

### 3.5. Application Concept

**Figure 14:**
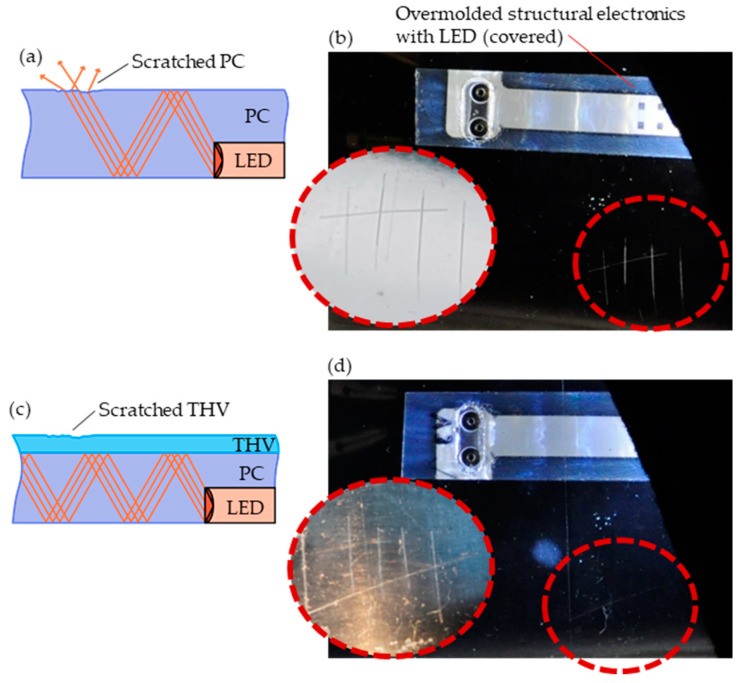
Sketches and images of two Smart@Surface [39] structural electronics demonstrators comprising an LED overmolded with PC. Light is coupled out if the PC surface is scratched (**a**,**b**). This can be avoided by adding a THV film serving as a cladding layer that protects the optical interface (**c**,**d**).

We demonstrated the relevance of the proposed approach for light guiding in an in-mold electronics application which is part of the “Smart@Surface” project [39]. Two concepts of waveguides, one without and one with cladding layers, were qualitatively compared. Here, light guiding was realized through an LED overmolded with a PC. Light is coupled out (and thus “lost”) in areas where the PC surface—possibly during its lifetime—becomes scratched, as illustrated in Figure 14a,b. However, when a THV layer was added to the overmolding step to serve as a cladding layer, this mechanical protection protected the optical interface from scratches: Figure 14c,d show no light outcoupling despite the scratches on the THV surface. The THV film was $O_{2}$ plasma-treated and molded using the relations presented in the effects diagram of Figure 11. 

## 4. Conclusions

Human–machine interfaces (HMIs) with backlighted touch keys and sliders require efficient waveguiding to be protected from environmental impacts. The low refractive index of the fluoropolymer THV makes this a suitable material for protecting a PC waveguide from environmental impact. While pristine THV films lack any adhesion with PC when overmolded using the IMD process, bonding can be established by treating the films first with $O_{2}$ plasma.

According to the XPS measurements, oxygen groups are created on the surface of the treated THV. Furthermore, the THV surface nanoscale roughness is increased considerably, as the AFM images show, manifesting in an increased water contact angle (WCA).

In our case, the plasma treatment duration, investigated between 0.5 and 3 min, did not substantially affect the PC–THV bonding strength investigated in peel tests. However, the adhesion-enabling effect of the plasma treatment diminishes with time: Peel loads on the treated THV films stored for 24 h before overmolding were half that of films overmolded already 1 h after treatment. This time dependency could not be related with the WCA, which stayed constant during this period.

The driving mechanism for the adhesion between the $O_{2}$ plasma-treated THV and the injection-molded PC remains elusive at this point. Potentially, the PC melt at high (injection) pressures can—contrary to water at ambient pressures and temperatures—bond tight with the surface of the plasma-treated THV. The formation of hydrogen bonds between the oxygen groups of the plasma-treated THV and the PC might then facilitate the bonding. The decrease in plasma treatment-created -OH and -OR groups with time is well documented in the literature (“aging” effect [16,17]). This would explain the lower peel loads observed for THV films stored before overmolding. Further research is necessary to find easier-to-scale and longer-lasting surface activation techniques than the proposed $O_{2}$ plasma treatment procedure. This will improve the applicability of the proposed approach in the backlighting of HMIs.

The influence of the molding parameters, namely the packing pressure, injection speed, melt temperature, and mold temperature were investigated in a two-level full factorial DoE. Higher (set) mold and melt temperatures, faster injection speeds, and lower packing pressures were found to produce overmolded films that yielded higher peel loads.

The DoE was numerically simulated using injection molding simulation software to investigate the physical principles. Here, it was found that the PC–THV interface temperatures are similarly affected by the molding parameters as they are by the measured peel loads. Seemingly, the higher and the longer the temperatures prevail above the glass transition temperature ($T_{g}$) of the PC, the stronger the interface becomes.

While this can be—quite obviously—achieved using higher mold and melt temperatures, faster injection speeds also increase the melt temperature through shear heating. A higher packing pressure results in the longer pressurization of the cavity, which translates into a higher heat transfer coefficient (HTC) at the PC–THV interface. Consequently, the PC–THV interface cools faster, and the bonding is weaker.

From a molder’s perspective, with typical injection molding cooling rates in mind, a “the longer and hotter, the stronger the PC–THV bonding” approach seems to be a reasonable guideline.

## Figures and Tables

**Table 1:** Process parameters for plasma treatment using an Oxford plasmalab system 80 plus.

Pressure (mTorr)	Forward Power (W)	$O_{2}$ Flow (sccm)
40	100	50

**Table 2:** Used design of experiment (DoE), where setting S05 designates the center point (CtPt).

Setting	A: Packing Pressure (bar)	B: Injection Speed (cm³/s)	C: Melt Temperature (°C)	D: Mold Temperature (°C)
S01	300	30	340	60
S02	150	70	340	60
S03	150	30	310	60
S04	300	70	310	60
S06	300	30	310	120
S07	150	70	310	120
S08	300	70	340	120
S09	150	30	340	120
S10	150	30	310	120
S11	300	70	310	120
S12	300	30	340	120
S13	150	70	340	120
S14	300	30	310	60
S15	150	70	310	60
S16	150	30	340	60
S17	300	70	340	60
S05 (CtPt)	225	50	325	90

**Table 3:** Default heat transfer coefficients (HTC) used by AMI during the different stages in the molding cycle. The HTC_Detached_ is used when the pressure is zero.

HTC_Filling_ (W/(m^2^·K))	HTC_Packing_ (W/(m^2^·K))	HTC_Detached_ (W/(m^2^·K))
5000	2500	1250

**Table 4:** Surface element composition of the THV films determined by XPS.

Element	Pristine THV	$O_{2}$ Plasma-Treated THV
F 1s	61.8%	55.3%
C 1s	38.2%	42.3%
O 1s	-	2.5%

**Table 5:** *p*-values of the factors investigated in the ANOVA of the DoE (Table 2) with the transformed response according to Equation (5). Herein, A denotes the packing pressure; B denotes the injection speed; C denotes the melt temperature; and D denotes the mold temperature.

Factor	*p*-Value	Factor	*p*-Value
A	0.003	B	0.000
C	0.000	D	0.000
CtPt	0.234		

## Data Availability

Not applicable.
